# Learning from the “tail end” of de-implementation: the case of chemical castration for localized prostate cancer

**DOI:** 10.1186/s43058-021-00224-8

**Published:** 2021-10-28

**Authors:** Ted A. Skolarus, Jane Forman, Jordan B. Sparks, Tabitha Metreger, Sarah T. Hawley, Megan V. Caram, Lesly Dossett, Alan Paniagua-Cruz, Danil V. Makarov, John T. Leppert, Jeremy B. Shelton, Kristian D. Stensland, Brent K. Hollenbeck, Vahakn Shahinian, Anne E. Sales, Daniela A. Wittmann

**Affiliations:** 1 VA HSR&D Center for Clinical Management Research, VA Ann Arbor Healthcare System, Ann Arbor, MI USA; 2 Department of Urology, Dow Division of Health Services Research, University of Michigan Medical School, Ann Arbor, MI USA; 3 Department of Internal Medicine, University of Michigan Medical School, Ann Arbor, MI USA; 4 Rogel Cancer Center, Michigan Medicine, Ann Arbor, MI USA; 5 VA New York Harbor Healthcare System and NYU School of Medicine Departments of Urology and Population Health, New York, NY USA; 6 Surgical Service, VA Palo Alto Health Care System, Palo Alto, CA USA; 7 Department of Urology, Stanford University, Stanford, CA USA; 8 VA Greater Los Angeles Healthcare System, Los Angeles, CA USA; 9 Department of Learning Health Sciences, University of Michigan Medical School, Ann Arbor, MI USA

**Keywords:** Implementation, Behavior change, De-implementation, Low-value, Intervention, Behavioral theory, Complex health interventions

## Abstract

**Background:**

Men with prostate cancer are often treated with the suppression of testosterone through long-acting injectable drugs termed chemical castration or androgen deprivation therapy (ADT). In most cases, ADT is not an appropriate treatment for localized prostate cancer, indicating low-value care. Guided by the Theoretical Domains Framework (TDF) and the Behavior Change Wheel’s Capability, Opportunity, Motivation Model (COM-B), we conducted a qualitative study to identify behavioral determinants of low-value ADT use to manage localized prostate cancer, and theory-based opportunities for de-implementation strategy development.

**Methods:**

We used national cancer registry and administrative data from 2016 to 2017 to examine the variation in low-value ADT use across Veterans Health Administration facilities. Using purposive sampling, we selected high- and low-performing sites to conduct 20 urology provider interviews regarding low-value ADT. We coded transcripts into TDF domains and mapped content to the COM-B model to generate a conceptual framework for addressing low-value ADT practices.

**Results:**

Our interview findings reflected provider perspectives on prescribing ADT as low-value localized prostate cancer treatment, including barriers and facilitators to de-implementing low-value ADT. We characterized providers as belonging in 1 of 3 categories with respect to low-value ADT use: 1) never prescribe 2); willing, under some circumstances, to prescribe: and 3) prescribe as an acceptable treatment option. Provider *capability* to prescribe low-value ADT depended on their knowledge of localized prostate cancer treatment options (*knowledge*) coupled with interpersonal skills to engage patients in educational discussion (*skills*). Provider *opportunity* to prescribe low-value ADT centered on the *environmental resources* to inform ADT decisions (e.g., multi-disciplinary review), perceived guideline availability, and *social roles and influences* regarding ADT practices, such as prior training. Provider *motivation* involved *goals* of ADT use, including patient preferences, *beliefs in capabilities/professional confidence*, and *beliefs about the consequences* of prescribing or not prescribing ADT.

**Conclusions:**

Use of the TDF domains and the COM-B model enabled us to conceptualize provider behavior with respect to low-value ADT use and clarify possible areas for intervention to effect de-implementation of low-value ADT prescribing in localized prostate cancer.

**Trial registration:**

ClinicalTrials.gov, NCT03579680

**Supplementary Information:**

The online version contains supplementary material available at 10.1186/s43058-021-00224-8.

Contributions to the literature

- De-implementation of low-value care has become a high priority, though rigorous approaches to de-implementation are understudied. Our findings uniquely conceptualize the spectrum of early to late, i.e., tail end, de-implementation, and relevant implications.
- Integrating our qualitative findings into a conceptual model of provider categories for de-implementation of low-value prostate cancer care, ranging from never use to offer as an acceptable treatment option, adds clarity to the specification of target audiences for behavior change strategies addressing low-value care.
- By using the TDF behavioral framework and COM-B model, we systematically identified opportunities for de-implementation strategy development, tailoring, and future comparative-effectiveness testing.

## Introduction

Increasing awareness of low-value care (i.e., care with little to no benefit, potential harm, and cost) in healthcare delivery systems has sparked significant interest in de-implementation. Effective de-implementation of low-value care, defined as reducing or stopping the use of a low-value health service provided to patients, has the potential to improve patient safety and outcomes and even decrease healthcare spending [1–4]. For example, the Choosing Wisely® campaign proposed over 400 recommendations to limit low-value care across 26 medical specialties, including cancer care [5–8]. Effectively implementing these and other recommendations to curb low-value care requires that we understand the science and practice of de-implementation.

De-implementation of low-value practices has a significant potential positive impact because low-value cancer care can adversely affect patient quality of life by exposing patients and caregivers to physical, emotional, and financial harms [8]. However, cancer care is unique when it comes to de-implementation as perceptions regarding rationing of cancer treatment may be psychologically, socially, and politically charged. Yet, because prostate cancer is the most common cancer among men, reducing its low-value care is important not only for patients at risk of overtreatment but also for public health more broadly. There have been, in fact, several major attempts to decrease low-value prostate cancer care by protecting patients with prostate cancer from overtreatment, ranging from recommendations to halt screening [9] to embracing active surveillance over definitive treatment given the limited 10-year survival benefits and significant treatment harms including incontinence, impotence, and bowel dysfunction [10].

Our work to understand and address low-value prostate cancer care centers on chemical castration and suppression of testosterone for localized disease using injectable drugs termed “androgen deprivation therapy” (ADT). We consider the use of ADT as a primary treatment for localized prostate cancer to be “low value” because neither long-term studies nor current guidelines support it and because it is associated with a spectrum of potential harms (e.g., osteoporosis, cardiac disease, diabetes) [11]. For example, the 2018 National Comprehensive Cancer Network Prostate Cancer Guidelines indicated “ADT should not be used as monotherapy in clinically localized prostate cancer.” [12] The most recent guidelines acknowledge ADT monotherapy may be used in settings where there are contraindications to definitive local therapy and limited life expectancy [13], but ADT use for localized prostate cancer treatment is not routinely recommended. In light of growing knowledge of such harms and policies to limit financial incentives for prescribing ADT [14], low-value castration practices for localized disease have decreased but still remain. In fact, low-value ADT is not infrequently prescribed to older patients with comorbidities with even less to gain from its ineffective use [15, 16].

Given the fact that de-implementation of low-value castration practices has already significantly decreased, we may be, to coin a phrase, at the “tail end” of de-implementation of low-value castration for localized prostate cancer. This end of the de-implementation spectrum creates an opportunity to learn how to best de-implement low-value ADT, allowing us to also discern how to de-implement other harmful practices for indolent localized cancers that have declined, yet remain in use. Further, if the drivers of tail-end de-implementation can be identified, the cost and value of such efforts can be better estimated. Lastly, it may also highlight unusual clinical scenarios that compel clinicians to choose this treatment for patients with localized disease. Indeed, better understanding the behavioral determinants of de-implementation not only informs strategy development to optimize prostate cancer care, it also adds to generalizable knowledge when it comes to de-implementation of ongoing low-value care for other cancer types (e.g., colon, skin, breast).

For these reasons, we conducted a qualitative study to identify the behavioral determinants of persistent low-value castration use with ADT to manage localized prostate cancer. We used the Theoretical Domains Framework (TDF) [17–19] and the Behavior Change Wheel’s Capability, Opportunity, Motivation Model (COM-B) [20] to explore urology provider barriers and facilitators with respect to providing low-value ADT. We characterized provider practices with respect to low-value ADT use for localized prostate cancer, ranging from those who never prescribe ADT to those who recommend ADT as an acceptable treatment option for localized prostate cancer, adding clarity to target audiences for behavior change and de-implementation strategies to curb incident and ongoing low-value castration practices. Our overarching goal was to inform and develop a conceptual model enabling us to design interventions to further reduce the use of low-value castration for prostate cancer survivors.

## Methods

### Study design

We conducted a qualitative descriptive study [21] using the TDF [17–19] and the Behavior Change Wheel’s COM-B to guide our data collection and analysis [20]. We selected this approach for two primary reasons. First, the TDF provides a comprehensive taxonomy of theoretical constructs related to behavior change, allowing us to organize the findings with respect to provider determinants of low-value ADT use to manage localized prostate cancer. Second, pairing our TDF findings with the COM-B model allows for the identification of subsequent behavioral theory-based opportunities for de-implementation strategy development and tailoring to address ongoing low-value castration practices. More specifically, pairing our TDF and COM-B findings provides a guidepost for identifying potential target behaviors that need changing to effectively minimize low-value castration. This is consistent with the Behavior Change Wheel methodology [20].

### Sampling and recruitment

We used the Veterans Affairs Corporate Data Warehouse database to identify national Veterans Health Administration facilities and their ADT utilization rates for men with incident, (new-onset) localized prostate cancer. Next, we purposively selected facilities with the highest and lowest rates of ADT use as primary treatment for localized prostate cancer, i.e., low-value castration. In our interview sample, rates of low-value ADT use ranged from 8.7 to 21.7% for high sites and from 0.8 to 1.9% among low sites. Additional details of our sampling and recruitment strategy are described in our published protocol [22]. Briefly, we obtained approval from medical center directors and urology section chiefs before contacting individual providers who order ADT, including urologists and urology advanced practice providers, at these facilities. After receiving approval, the members of the study team emailed providers with information about the purpose of the study and a brief description of the telephone interview questions. We consented interested urology providers over the phone or through email at which time we also scheduled interviews with our qualitative researchers.

### Data collection

Consistent with prior work [23], our research team used the TDF to inform interview guide development and to help frame our understanding of urology provider attitudes about prescribing ADT with a particular focus on low-value use for the treatment of localized disease. Our interview guide initially asked about provider behavior vis-a-vis patients with comorbidities who had intermediate-risk prostate cancer (unspecified) and no evidence of cancer spread who would be eligible for definitive treatment but might not prefer it. We also asked questions about provider approaches to the use of ADT in the more general setting of localized prostate cancer, regardless of risk. The guide included the following domains: urologists’ typical treatment for localized prostate cancer, professional perceptions of treatment with ADT, and interventions to decrease the use of low-value ADT (see Additional file 1: Appendix). We pilot tested and refined the guide among our study team, which included 5 urologists (TS, BH, JS, JL, DM); a medical oncologist (MC); 3 doctoral researchers, 1 with expertise in decision-making (SH) and 2 with expertise in qualitative methods (JF, DW); and a masters-level researcher trained in qualitative methods (JS). Interviewees were told that we were interested in understanding the variation in the use of ADT for prostate cancer. The interviews then proceeded with a patient vignette; interviewees were asked to discuss how they would go about formulating their treatment plan. Three members of the research team (JF, JS, DW) conducted a total of 20 urology provider interviews (19 urologists, 1 urology nurse practitioner), 17 at high-frequency low-value ADT sites, and 3 at low-frequency low-value ADT sites. Urology providers were based at facilities in 12 states representing all major US regions (e.g., Midwest, South, Pacific Northwest). Provider interviews were conducted by telephone and lasted an average of 29 min. Using a similar approach, we also identified and interviewed 12 patients treated with ADT for their localized prostate cancer across 6 high-frequency low-value ADT sites. We modified our interview guide for the patient interviews to examine their understanding of the treatment options, provider recommendations, and the side effects of ADT. This project was approved by the VA Ann Arbor Healthcare System Institutional Review Board.

### Data analysis

All provider interviews were audio-recorded and transcribed verbatim. We used a deductive qualitative content analysis approach [24] in which the coding scheme comprised TDF domains, organized in the COM-B model (Table 1). In using the TDF domains to analyze the content of the interviews, we anticipated interview findings would naturally indicate how these TDF domains would act as barriers or facilitators. Four research team members (DW, JF, JS, TS) independently coded at least 5 interview transcripts using these codes and, in an iterative process, developed project-specific definitions of each code. Overall, at least two team members coded each transcript and resolved all discrepancies through a discussion at regular coding meetings.

**Table 1:** COM-B and TDF domains relevant to low-value ADT use for localized prostate cancer

COM-B domain	TDF domain	Definition [20,23]
Capability—psychological	Knowledge	An awareness of the existence of something
	Skills (interpersonal)	An ability or proficiency acquired through practice
	Decision processes	The ability to retain information, focus selectively on aspects of the environment, and choose between two or more alternatives
	Behavioral regulation	Anything aimed at managing or changing objectively observed or measured actions
Opportunity—physical	Environmental context and resources	Any circumstance of a person’s situation or environment that discourages or encourages the development of skills and abilities, independence, social competence, and adaptive behavior
Opportunity—social	Social influences	Those interpersonal processes that can cause individuals to change their thoughts, feelings, or behaviors
Motivation—reflective	Professional/social role and identity	A coherent set of behaviors and displayed personal qualities of an individual in a social or work setting
	Beliefs about consequences	Acceptance of the truth, reality, or validity about outcomes of a behavior in a given situation
	Intentions	A conscious decision to perform a behavior or a resolve to act in a certain way
	Goals	Mental representations of outcomes or end states that an individual wants to achieve
Motivation—automatic	Emotion	A complex reaction pattern, involving experiential, behavioral, and physiological elements, by which the individual attempts to deal with a personally significant matter or event

Our selection of significant factors that influenced provider behavior and the conceptual model was an interim product of our analysis. First, as interviewers and analysts, we were immersed in the data. Second, we started coding the data by using all of the TDF domains and found the selected constructs were prominent and the best fit for the data. Next, we developed the conceptual model through the inductive process, verifying and refining it as our analysis progressed. All relevant data fit into the existing TDF codes. After the data were coded, we used the NVivo 11™ qualitative software to organize the coded data. Findings were then organized into TDF domains and presented as barriers and facilitators. Based on these findings, our team then finalized a conceptual model for low-value ADT use structured around the TDF and COM-B domains. While subsequently conducting the patient interviews, our team discovered early thematic saturation with respect to the use and side effects of ADT, with strong deference to provider decisions regarding ADT prescribing consistent with prior prostate cancer treatment decision-making literature [25, 26]. For these reasons, we did not conduct a formal qualitative analysis on patient interview data.

## Results

**Fig. 1:**
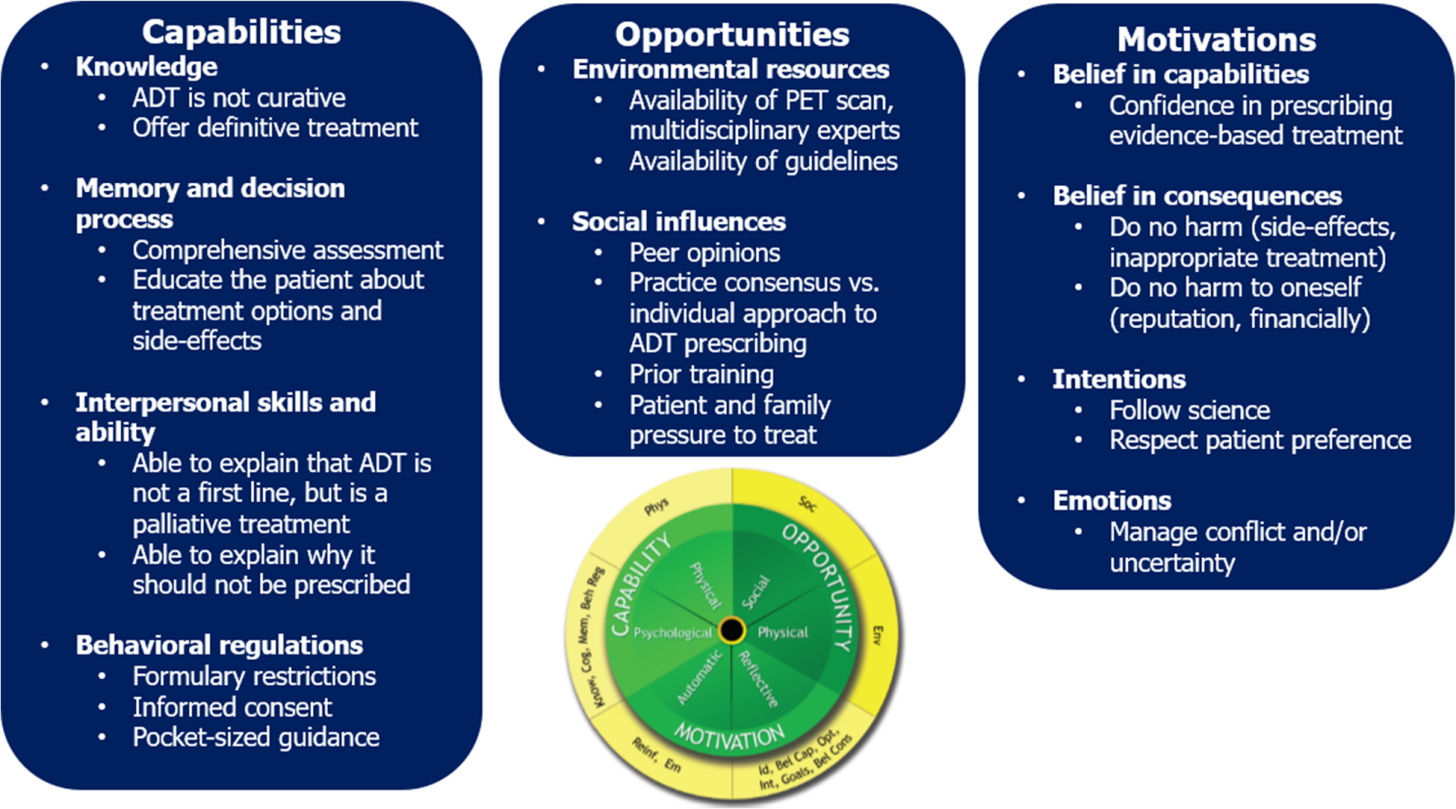
Behavior change wheel’s COM-B [20] and TDF domains for low-value ADT as localized prostate cancer treatment

Our interview findings reflected provider perspectives on prescribing ADT as a low-value localized prostate cancer treatment. As shown in Table 1 and illustrated in Fig. 1, we characterized the findings according to the COM-B domains in order to understand the low-value ADT prescribing behavior. This allows for conceptualization of future contributing factors that can facilitate or obstruct behavior change, and functions needed to support the de-implementation of low-value ADT. For *capability*, our data were best characterized according to psychological capability and the TDF domains of knowledge, decision process, skills (interpersonal), and behavioral regulation. For *opportunity*, we found provider responses corresponding to both physical and social opportunity and TDF domains of environmental context and resources, and social influences. Lastly, for *motivation*, the primary findings centered on reflective motivation and the TDF domains of beliefs about consequences, intentions, and social and professional role and identity, with mentions of automatic motivation related to emotion. Relevant COM-B and TDF domains are further described below with supporting provider quotes.

### COM-B domain: capability

#### TDF domain: knowledge

Providers uniformly recognized ADT was not a curative treatment.

So I think there’s plenty of data out there that shows that ADT is not curative. So it delays the progression of disease. (011)

So I mean, ADT is absolutely not curative at all. . . I basically tell them, it can kind of put the fire out, but the fire is still there, it’s just smoldering. (015)

In general, urologists suggested patients with localized prostate cancer be recommended primary treatments with guideline-recommended definitive therapy, such as surgery, radiation, or radiation in combination with hormonal therapy. For patients averse to definitive treatment, providers typically suggested offering active surveillance with PSA testing, with or without follow-up biopsies and imaging to monitor for cancer progression and/or metastases.

I want to pursue active surveillance, . . . I would recommend repeat PSA checks, generally every six months. If there was a concern in the trend or the doubling time or velocity, then I might move that up a little bit before 6 months, depending on when the patient’s last biopsy was and I would make sure that there was a biopsy on the horizon, and if the patient had had an MRI or not previously, I would take that into consideration and then consider repeating the MRI or performing one for the first time as well on a patient in active surveillance. (002)

In some cases, providers suggested alternative treatment strategies like cryotherapy or high-frequency ultrasound ablation (HIFU) for patients averse to commonly recommended treatments.

I still would think that I would encourage the patient to get all the information and find out why it is that he is against surgery and radiation, . . . And if he wanted something a little bit off the reservation I would talk to him about HIFU or cryotherapy. (002)

One provider highlighted the case of an elderly male with limited life expectancy for whom ADT for localized disease might be acceptable according to the most recent NCCN guidelines.

You know it’s, I mean I might be a little more prone to do it in that 94-year-old because he’s not a candidate for anything else. (001)

Providers also expressed recognition of the deleterious effects of chemical castration with ADT on patients’ lives.

So if they want to maintain their sexual activity, you know, administering androgen deprivation therapy will definitely disallow that. (013)

. . . I mean initially most of them are concerned about the hot flashes. And they’re concerned about the loss in sexual function. I would say those are two biggest things. . . (012)

Despite our purposive sampling strategy to include providers at sites with high rates of low-value ADT use, only one provider expressed a willingness to treat localized prostate cancer with ADT as the first-line therapy.

And if he is not, if he expresses no interest in either surgical intervention or any forms of radiation, you know, you need to have the discussion whether or not this patient understands the nature of the disease, which it likely will be progressive. And that there is (sic) also alternatives such as androgen deprivation therapy, either intermittent therapy versus continual therapy. (015)

While most urology providers in our sample appeared to have a strong knowledge base regarding ADT use and its harms, at least one demonstrated a potential lack of knowledge of the inappropriateness of prescribing ADT for localized disease (015). As a result, Knowledge appears to have been a facilitator of stopping ADT, but lack of knowledge in at least a small proportion of providers can be a barrier to de-implementation among those continuing to recommend low-value ADT.

#### TDF domain: decision process

All providers described their decision process as starting with a comprehensive evaluation of the patient and prostate cancer severity. In order to make treatment recommendations, providers said PSA level and rate of doubling time, biopsy results, the patient’s age, comorbidities, and the patient’s goals should be assessed. They discussed referring to guidelines in the process.

…what are his other health risks? Does he have cardiovascular disease? Does he have severe COPD? What’s his life expectancy? And then what are his goals? We have patients who just adamantly don’t want anything done, or they’re afraid of the outcomes of surgery or radiation and they don’t want to lose some of their virility or they’re afraid of incontinence…you always have to ask the patient what their goals are and look at the patient overall. And if they’re a good 72 versus a 72-year old that may have multiple comorbidities and doesn’t have a life expectancy beyond the next few years. I think you just have to kind of tailor it to each individual. (012)

…treat everybody with localized disease with local therapy. But you were right when I have a patient who refuses all the localized treatments I only reserve hormonal therapy for a [unintelligible 10:36] doubling time, extremely high-risk patients. (014)

All providers noted that educating the patient about the treatment options and the side effects of treatment was a necessary aspect of their decision making, but there was a range in the thoroughness of the education described in interviews from discussions of risks and benefits to providing a brochure on treatments and side effects.

…you just have to give him informed treatment decisions and get the risk benefits of each of these things. Because sometimes they come in with false ideas on what these treatments entail...What you’re trying to do is give them the most information possible to make that decision. (001)

We actually have a brochure…that talks about prostate being removed, radiation beam, or the radiation seeds. And then we literally cover each one of those with these potential side effects…we actually go through and give those, the choices. And then we do talk about no treatment and what that means for their mortality. And then we also talk about active surveillance and who actually fits the requirements for active surveillance. (013 – note this quote indicates more thorough education than most other providers so may not be representative)

Several providers said explicitly that sharing the treatment decision with the patient was an important goal for them.

I absolutely take patient preference into consideration and I think that ultimately treatment decision making should be shared. (002)

…in my experience if that patient is a healthy patient who is, you know, has a 10, 20 life year expectancy then a lot of times that preference is based on misinformation and I think that patient education and that shared decision-making model can help them. And it’s not that difficult for most patients, to guide them to kind of, back on to course. (019)

While most providers were very clear that ADT was not the first-line therapy for localized prostate cancer, they were more nuanced and described a range of approaches when discussing seeing a new patient who had already been prescribed ADT by another physician. Some did not want to criticize the previous provider by de-implementing ADT, and others thought that if the patient was happy on ADT, they should not make changes.

So when somebody has sort of been on a treatment plan, I don’t try to destruct it because obviously that’s what the patient wanted. He is content with it. It is controlling his cancer. I mean there’s that benefit. I don’t disregard that . . . I’m not going to rock the boat. (007)

Some thought of an intermediate step—they described moving the patient towards intermittent therapy would reduce the harms of the ADT.

I think again, if they’ve been happy with how they’re doing, I would continue that (ADT). I’d give them the benefit of doing intermittent therapy. I think that has some benefit, to, again, to decrease side effects . . . (016)

But some said that they would educate the patient about ADT inefficiencies and harms and would, in the context of developing a trusting patient-physician relationship, recommend discontinuation; they would do so repeatedly if necessary.

I just put a positive spin on it and say hey, you know, you’ve been doing this every three months, things have been looking really good, your numbers are looking great, we need to repeat your scans just to make sure. But if your scans come back and it’s not showing anything, and you really don’t have any metastatic disease, then the newest research out there. . . and this is how I do the whole discussion with them. That the newest research is saying that we may be able to use this medicine longer if we don’t use it every three months . . . (013)

Although most providers said ADT was not the recommended treatment for localized prostate cancer because curative treatments exist and ADT is not curative, a plurality was willing to consider prescribing ADT treatment in certain circumstances. Some cited practical patient preferences, such as accomplishing other goals before treatment or travel.

And then I just gave another scenario where if the patient really did want to have treatment with surgery or radiation but just could not at that time because they are maybe addressing another medical problem or some people are taking care of another family member and so they couldn’t do it, but they were worried that it may progress until they get a chance to have the definitive treatment, I may offer them hormone therapy in theory just to prevent it from progressing until they got the definitive treatment, that they want it. (003)

One provider thought that ADT could delay metastases when the PSA level was high but recognized that this was not an evidence-based approach.

You’ve identified a patient who has a high risk of cancer metastasis, and the thought is, well, maybe you can initiate a treatment to avoid or delay metastasis, which really would be, if local treatment is out of the question, maybe a systemic treatment would be beneficial there. But on the flip side, there’s no great data to support that decision. So it depends on if you’re mostly a data-driven person or if you kind of want to shoot from the hip a little bit, that could influence your decision one way or the other. (008)

Only one provider reported that he had taken patients off ADT because he disagreed with the previous provider’s treatment approach. Urologists’ efforts to educate patients about low-value ADT clearly facilitate the process towards optimal care. However, an unwillingness to criticize a previous provider or reluctance to “rock the boat” if a patient is satisfied with his ADT care can be considered a barrier in the decision-making process, a barrier potentially ameliorated by providing providers with talking points or discussion guides.

#### TDF domain: skills

Several providers described how they would educate the patient about the value and harms of ADT and other treatment options in a way that demonstrated sensitivity to the patient’s need to understand what was involved. They took pride in being able to speak to the patient’s worries by being straightforward and practical demonstrating interpersonal skills and ability to convey complex medical information in an easily accessible way that patients could understand. Provider explanations were detailed (e.g., guidelines, considerations of the patient’s comorbidities and type of cancer, how ADT works, treatment outcomes—side effects, mortality), with attention to making the information accessible to patients through using plain language. This suggests they did not spare time trying to help the patient understand the decision they would be making regarding initiating or continuing ADT.

We give them the NCCN guidelines. And so we actually have a brochure that comes from Krames, K-R-A-M-E-S. It’s an educational brochure that we use that talks about prostate being removed, radiation beam, or the radiation seeds. And then we literally cover each one of those with these potential side effects. And if somebody does not meet the criteria for maybe the prostate being removed because, you know, their hearts are too bad or their too old or, you know, major issues with their other health issues. Versus maybe they can’t have the seeds because their prostate size is way too large for it or it’s too aggressive on their cancer. But we actually go through and give those, the choices. And then we do talk about no treatment and what that means for their mortality. And then we also talk about active surveillance and who actually fits the requirements for active surveillance. (013)

…I try to educate them on exactly what hormone therapy does. And I try to put it into layman’s terms, such as, you know, testosterone is like gasoline for a car, but eventually the prostate cancer gets smarter and figures out how to live without it. So, I think putting it into more relative terms for the patients can really help them understand the fact that it is not a cure. (021)

Providers’ responses indicated that having interpersonal skills and the ability to clearly relay complex medical information can facilitate a productive discussion of de-implementation of low-value ADT with the patient.

#### TDF domain: behavioral regulation

We discovered a variety of opinions among providers about how to accomplish the de-implementation of low-value ADT. Despite our purposive sampling of facilities with higher low-value ADT rates, the majority of providers supported decreasing low-value ADT and offered insights into addressing residual, tail-end low-value ADT practices. For example, several providers thought having a concise guide with talking points would be helpful (e.g., script), for example:

If I wasn’t seeing a lot of prostate cancer patients, then it might be nice to have some sort of quick reference about when I should be giving hormones. What are the indications for androgen deprivation therapy? You know, in a kind of concise, user friendly way. Perhaps it’s also beneficial to have a nice canned, like a canned talk about what are the risks of the therapy, like a very easy summary with eight bullet points about here are the things that could go wrong with hormones. Here are the things you need to be most concerned about in a very bite-sized way. That could be useful. (008)

We interpreted this to mean that when in doubt about providing ADT care, particularly to a patient already established on low-value ADT, it would help to have a quick reference to remind oneself about why this might not be the best option for the patient.

One provider suggested that exposure to how others practice (i.e., audit and feedback) would help change behavior. He gave the Michigan Urologic Surgery Improvement Collaborative (MUSIC) as an example in which unnecessary imaging decreased when MUSIC published variation in imaging [27].

And I think even utilizing MUSIC as a platform. You know I think there’s something like 80-90% of the practices, the urology practices in [State] are participants in MUSIC… And you know, their website is open to all as far as I understand it. So I think like I said, it has been a great platform. And I think their method of really keeping things straight forward, very simple, I think it has been very effective. (011)

While most resisted the idea of a formulary restriction, at least one participant thought that having someone who oversees cases to evaluate the appropriateness of care might be helpful.

I think that’s a little bit too much restriction on the practitioners. Actually, I don’t think they will like it… (014)

In the VA hospital, perhaps you can have the checks and balances of the pharmacist getting involved, but that’s one of the things that drives VA doctors absolutely bananas. . . But from the system standpoint, having a check against the prescribing practices could be useful. It would be very painful to have that. . . . . And so, perhaps, I mean there’s a way to order the medicine in such a way that it forces you to order it for an indicated purpose only. You know, you have to click a box. Yes, I’m giving radiation, yes, this patient has metastatic diseases. Yes, this patient has biochemical recurrence, whatever. And if you don’t meet one of those criteria, then you cannot order the drug. (008)

One practitioner described practicing in a centralized clinic where the appropriateness of ADT injections is assessed regularly as a method of quality assurance and internal peer-review.

But what we try to do in our clinic at least is to kind of centralize hormone injection care so that . . . when patients are coming on one day for hormone injections, and one person is overseeing all those injections to make sure they’re all appropriate and so forth, which we have the luxury of because we have people here who understand when you should and shouldn’t give hormone therapy. (008)

While the institution of informed consent documentation was considered by some as impractical, one provider thought that it would force a discussion of cancer risk and treatment side effects which would be beneficial.

. . . I mean, we’re essentially getting informed consent by telling them the risks and benefits and side-effects of it. So I don’t’ think a formal informed consent is necessary. (017)

I think it’s valuable to do it (IC). I, again, don’t know that signing a computer form or paper form is a valid way to confirm that someone really understands what they’re getting into . . . but I think if you verbally talk about it and document that, that’s probably better than signing a goofy little computer signature. (016)

Despite some reluctance on the part of the providers, there seemed to be a consensus that some form of monitoring or guidance about when not to use ADT or even restriction could be beneficial in clinical care. A potential barrier to de-implementation was the lack of standardized strategies. If we accept most providers were open to have support for de-implementation, it was also clear that one size does not fit all. A successful approach would potentially need to include a menu of strategies so that individual providers could select one that would suit personal preference and the characteristics of a particular patient.

### COM-B domain: opportunity

#### TDF domain: environmental context and resources

The majority of providers mentioned referring to guidelines, for example, provided by the American Urological Association [28], National Comprehensive Cancer Network [10], Michigan Urological Surgery Improvement Collaborative [29], published randomized trials, and other resources (e.g., inserts from pharmaceutical companies, life prediction tables).

… I rely on data from clinical trials, from national guidelines, from even expert opinion when there’s no better evidence, but I try to refer to the literature as much as possible. That’s just my own practice. (008)

So I use the AUA guidelines quite a bit. I use the NCCN guidelines. Those are probably my two main resources. And I guess within [State], you know, there’s some guidelines set for us by the MUSIC group. So I guess those are the, my probably three main resources. (011)

Providers valued having an opportunity to consult with multidisciplinary, fellowship-trained colleagues.

We as a team went together and discussed this whole thing and kind of hashed it out. So everybody, while it’s a change, we’re going to try and follow and have the guidelines to be able to back us up; so that’s what we’re doing. (013)

… I think we’re very influenced by our peers that’s why we have weekly educational meetings to talk about cases just, I mean very similar to what we’re talking about. I mean, we bring up current studies. We bring up past studies. (015)

A majority of providers suggested that being in touch with cutting-edge guidance either through guidelines or their peers did and would facilitate the provision of optimal care. However, it was not clear that the availability of a multidisciplinary team was a resource in every VA facility or available to all the participants in the study. This lack of environmental resources was a potential barrier to de-implementation.

#### TDF domain: social and professional role and identity

The majority of providers described themselves as managing patients treated with ADT, but their roles in ADT prescription and administration varied. While several providers stated ADT was commonly prescribed and administered by urology, just as many providers said they referred all patients who they thought needed ADT to medical oncology, or when a patient’s cancer shifted from being localized to metastatic.

It’s actually pretty common for us at the VA . . . we would see them, we would make the diagnosis of prostate cancer and then we would probably initiate their ADT. And it’s not uncommon for us to be the ones who kind of give the actual shot, Lupron or whatever, going forward. (006)

So here at the VA, we actually aren’t the primary prescribers of ADT. If we think a patient is needed for ADT, we actually refer them to medical oncology, and they usually give the ADT. (017)

So we only really manage them up and to the point where they’re either hormone resistant or they’re metastatic. And then we send them to hem onc. (012)

Providers mostly agreed prescribing ADT for localized disease was inappropriate, again despite our purposive facility sampling. A few providers added that prescribing ADT in those instances was generally viewed as “old school” and not commonly done now.

We certainly talk about ADT, but we don’t usually talk about it in the primary setting. (002)

I’d say it’s perceived as old school. I think that’s one of the things that we have learned that was done fairly commonly, perhaps in the 80s and 90s, for a variety of reasons. And we have since learned that that’s not necessary. And I think that as more people are graduating residencies being taught that, then it’s hopefully being a thing of the past. I know it’s not completely a thing of the past. In fact, it’s probably not nearly as much as it needs to be. But hopefully that’s becoming kind of phased out as many practices in urology are, they get phased out over the course of years and years.

Social and professional identity can thus be viewed as a potential facilitator in the sense that at least some providers saw ADT care as the domain of medical oncology. Clarifying roles by seeing urologists as only treating localized disease with appropriate definitive treatments and referring for ADT care to medical oncologists for progressed or metastatic disease might be a facilitator in efforts to de-implement low-value ADT in localized prostate cancer.

#### TDF domain: social influences

Providers also described the influence of patients’ emotional challenges, such as fear of having cancer, reluctance to have definitive treatment, indecisiveness and deferral of definitive treatment, or family pressure to “do something” as barriers to stopping low-value ADT.

. . . I think they feel like they’re doing something. And they, because they have cancer, they got to do something. (016)

Yeah, I would only, if the patient was like really worried, like let’s say that they had another operation or something else, so they couldn’t do their prostate cancer definitive treatment and they were really worried that they were doing nothing about the prostate cancer while they were taking care of other medical issues, that’s the only time I would even consider it. (003)

Patients that are unsure of what they want to do and they haven’t made a decision and the doctor doesn’t want to do anything, so they’ll start him on ADT and say okay, you got three months to make a decision, you got six months to make a decision. So that’s commonly done, and that is fine. But most of the people that are on primary ADT, it’s simply because they were unable to say I want active surveillance or I want cryo, HIFU, surgery, radiation. So they’re still on the fence. You’ll find that people that are on the fence, the urologist gives them something, tells them to mull it over, and maybe he’ll get off that fence. But that’s it. (004)

Taken together, the social influences mentioned by providers tended towards barriers, i.e., supporting initiation and continuation of low-value ADT.

### COM-B domain: motivation

#### TDF domain: beliefs about capabilities

All providers, with the exception of a recent graduate, spoke with confidence about their ability to make a clear representation of treatment options to their patients. One provider mentioned using what they learned from prior experience with ADT to help guide treatment decision-making.

And I suppose there’s always a certain amount of within the confines of the guidelines using your own experience and what you know, so what I’ve done in the past and has worked and I take that into consideration as well. (002)

One provider described how he helps empower patients to make informed treatment decisions by providing them with information about available treatments based on their volume and type of prostate cancer.

Well, I mean, you know, it’s his choice. You know I’m not going to tell him this is what he has to do, he has to decide, but I’ll present based on the volume and they type of cancer, if it’s a 4+3 I would be encouraging him more to do something about it. And the radiation is actually very simple, and can kind of nip it in the bud, but if this is what he wants you’re going to be doing whatever he wants. In other words, it’s the patient’s choice, you just have to give him informed treatment decisions and get the risk benefits of each of these things. (001)

Several providers made statements to suggest they were comfortable applying their knowledge in determining an appropriate course of treatment for localized prostate cancer.

…I give them the data if they, of what are the best treatments available. The surgery and, but again, the surgery is not shown to be better than the high dose radiation. . . . But again, if that’s what they want then I’m still going to say, well that’s your choice. I mean I have some patients who have cancer who we started to get them ready for (radiation), we put them on hormones to downsize and sensitize the cells to radiation, and now he just doesn’t want to do anything. There’s not much I can do about it. I can tell them, you know, you really need to have something done because this could be a problem, but it is their choice always. (001)

Another expressed confidence in being able to change a patient’s ADT management (i.e., stopping hormone therapy), if it was determined that it was not an appropriate or necessary treatment.

…I’m not bashful about that. If I feel like someone has inappropriately treated somebody, I will not hesitate to say, hey listen, I just wanted to make sure I wasn’t missing anything. What was the reason for doing this? (008)

Providers’ confidence in their capabilities is a good example of a facilitator in the discussion of ADT de-implementation.

#### TDF domain: belief about consequences

Perhaps the biggest driver of the decision not to prescribe or reluctance to prescribe low-value ADT were providers’ beliefs about the consequences of this treatment: the fact that it was not curative could make the patient more resistant to ADT later and had harmful side effects. While we coded understanding of ADT treatment indications, guideline recommendations, and side effects in the knowledge domain, this domain was more relevant to decisions, consequences, and outcomes expectancies regarding continuing or discontinuing ADT in low-value situations.

…this guy is not wanting active treatment due to a combination of age and other comorbidities. So he’s gambling on the fact that he will die of some other natural cause way before he will ever develop significant metastatic disease and symptoms from that. Therefore, why would you want to give somebody a drug that probably has no benefit and will have a significant amount of side effects? So in other words, his quality of life will be worse. So the side effects of the disease are actually less than the side effects of the treatment. (004)

I think that, you know, after somebody has been on, I think that androgen deprivation in some cases can be worse than the disease itself. There are many things that it puts you at risk for, osteoporosis, hip fractures, which in men are much more, the mortality from a hip fracture in men is like three times out what it is in women. You know, the anemia, the cardiovascular effects, you know, the quality of life effects as well. (021)

Some emphasized the fact that comorbidities might be more life-threatening for an individual patient, especially if the patient was older.

…if they had coronary disease, that’s, you know, there’s articles out basically showing, yes it increased their risk for heart attack, no it does not increase their risk for heart attack. And you just have to present that it could be either way, with them, and if they’ve got bad, if they’re that fragile then you want to be, perhaps err on the side of caution that you don’t give them that. (001)

The consequences of treatment for the patient were front and center in urology providers’ accounts and can be considered as facilitators.

#### TDF domain: intentions

Providers clearly wished to walk the line between staying with the scientific evidence for their treatment recommendations and responding to patient preferences, in essence managing clinical indications and values-concordant decision-making. The balance of these two intentions was not the same for everyone. Some signaled they could not abandon science and therefore would take time, even several visits, to persuade the patient regarding low-value ADT use. Others stated clearly that if the patient wanted ADT after all the education, they would go along with the patient’s preference.

I start out by trying to educate the patient on, you know, kind of the basics of prostate cancer. And I try to personalize it for them, you know, by putting their [unintelligible 13:48] on their specific parameters into perspective with what’s accepted standard of care. And then certainly keeping their preferences at the top of the list. And so I don’t, you know, so if a patient is not agreeing to treatments, you know, you just do the best you can and support them along the way. (011)

The providers seemed to understand that their conversations with patients were complex, requiring both clinical knowledge and personal sensitivity. Their intentions were positive and served as facilitators of de-implementation. However, at times, the patient’s preference prevailed, leading to the providers’ provision of care that they did not think was optimal and became a barrier to the intended, optimal care.

#### TDF domain: emotion

A few providers were concerned about the consequences for themselves if they did not offer to the patient treatment that the patient desired.

Yeah, because this is America and they will come in and they, oh, you’re not treating my cancer? . . . the guy is going to find another doctor and then he’s going to write on the internet and say that that doctor is an idiot and he didn’t treat me for my cancer. . . there is no defending yourself on the internet . . . if the patient wants to be treated, or his wife wants to be treated, and if you don’t treat them, they’re going to drag their husband to the next guy down the street... you know, medicine is a business. (004)

These responses appeared to represent both the providers’ desire to accommodate the patient and desire to avoid getting into a disagreement with either the patient or the patient’s family in an emotionally charged cancer discussion. This is a barrier whose interpretation must be nuanced: the provider’s desire to avoid conflict is an emotional barrier for the provider who may not wish to have an unhappy patient. This barrier might be assisted with a discussion guide although a deeper, more psychological approach might be relevant to assist with behavior change.

#### Conceptual model for de-implementation of low-value chemical castration for localized prostate cancer

**Fig. 2:**
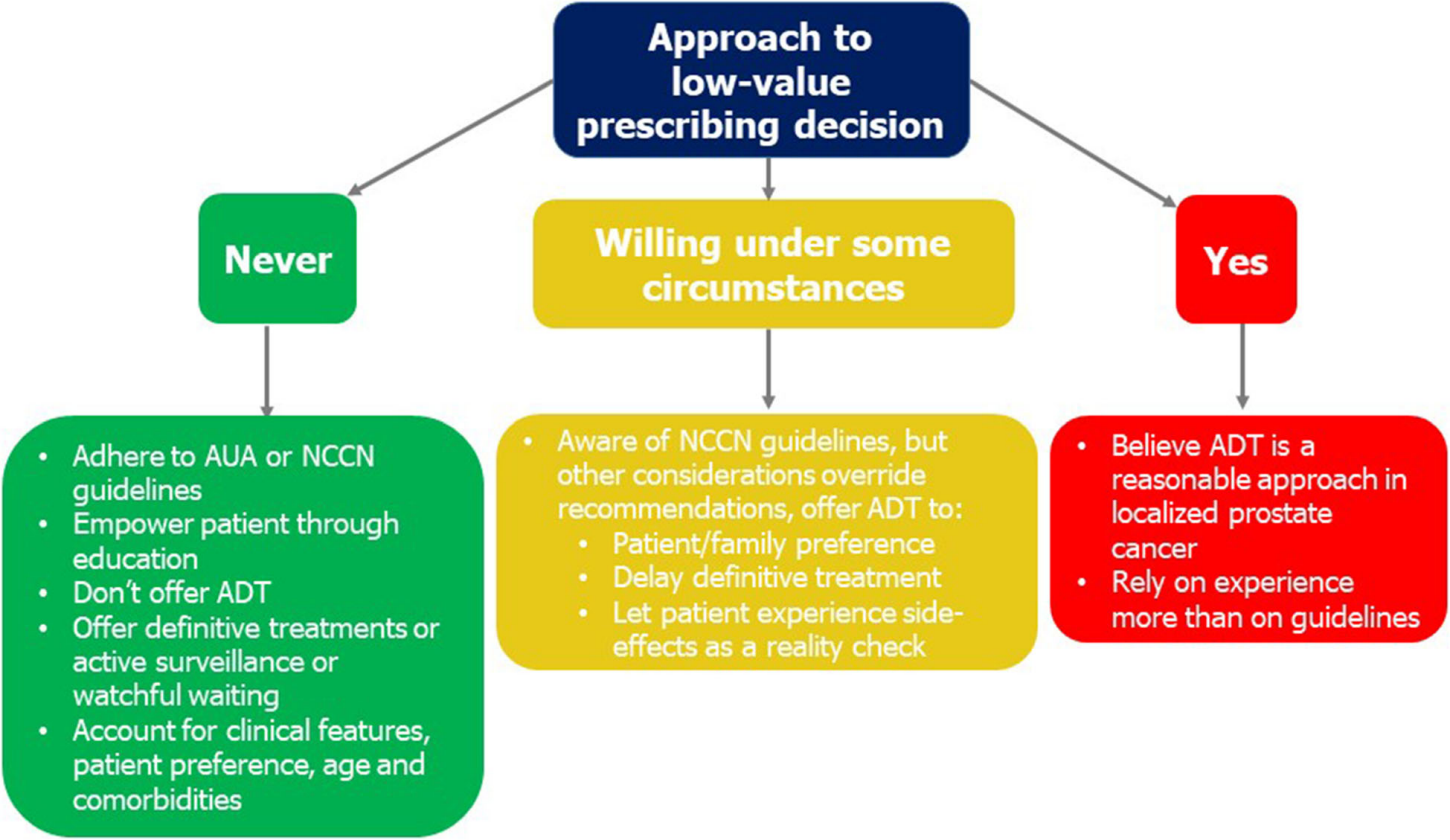
Conceptual model of provider categories for de-implementation of low-value prostate cancer care

We integrated our qualitative findings into a conceptual model to demonstrate providers’ experience and attitude towards low-value ADT prescribing. Informed by our findings, we characterized three types of provider practices with respect to low-value ADT use ranging from “never use” to “recommend as an acceptable treatment option” for localized prostate cancer treatment (Fig. 2). Among providers stating they never prescribed low-value ADT for localized prostate cancer, there was a strong tendency to cite guideline recommendations against ADT for this indication, to recommend definitive treatment with surgery or radiation therapy or surveillance strategies, to not recommend ADT as a primary treatment option, and finally to empower the patient through education of the side effects. Providers “willing to consider” ADT as primary treatment or continuing it among patients transferring to their practice also cited guidelines; they were open to prescribing ADT, however, based on strong patient or family preference after counseling about side effects. While this would be consistent with value-concordant decision-making, the weak clinical indication and side effects render the care low value. One went so far as to let patients experience the side effects such that they will ask to stop ADT. Providers willing to prescribe low-value ADT also cited unique clinical scenarios outside of guidelines where the practice might make sense (e.g., locally advanced disease with symptoms). The final and smallest group indicated ADT was a potential option based on their experience rather than guidelines.

## Discussion

This behavioral theory-based, qualitative descriptive study provides insights into barriers and facilitators discerned at the “tail end” of the process of de-implementation of low-value chemical castration with ADT as a treatment for localized prostate cancer and suggests potential approaches to discontinuing the practice altogether. By using the TDF behavioral framework and COM-B model, we were able to organize our findings into a conceptual model that aids understanding of thought processes leading to low-value ADT use and, in turn, helps identify opportunities for de-implementation strategy development and tailoring in later stages of de-implementation.

We found the majority of providers had the *capability* to de-implement low-value ADT. Facilitators of de-implementation were their knowledge of treatment options (*knowledge*), coupled with interpersonal, capable skills (*skills*) through which to educate patients about why ADT was not the optimal treatment choice and come to appropriate treatment decision (*decision-making process*) to not prescribe ADT. At the same time, there were barriers for some providers in these domains. These included the lack of knowledge in at least one provider, reluctance to act on the knowledge in the decision-making process when the patient or the family did not want to go along with the recommendation for fear of losing patients to other providers more willing to prescribe ADT. In addition, in some cases, providers did not have available support through which they could modify their prescribing behavior (*behavior regulation*) and had diverse opinions about what support would enhance their change in prescribing behavior.

Providers clearly described the *opportunity* to facilitate stopping prescribing low-value ADT for localized prostate cancer. The TDF domains most closely endorsed were resources (*environmental resources*), such as practice guidelines, the availability of multidisciplinary teams for case discussion, and collaborative approaches (*social influences*) where they could compare their own practice to the practices of others. The fact that these are, with the exception of practice guidelines, aspirational resources which do not exist in all VA practices represents a barrier in the current environment. Social influences also came in the form of patient or family pressure to provide or continue low-value ADT, serving as barriers to de-implementation.

The TDF domains in *motivations* describe the “human” element of prescribers’ behavior and are, of necessity, more complex. Facilitators of ending low-value ADT for localized prostate cancer patients were providers’ clear intentions (*intentions*) to provide the best, evidence-based care for their patients, to reduce the harm of side effects of ADT (*beliefs about consequences*), and confidence in their ability to either have an informed discussion or not prescribe low-value ADT in the first place (*belief about capabilities*). Their intentions were complicated by fear of losing patients (*emotion*). Providers indicated that at times, they preferred to avoid challenging their patients, ultimately for fear patients would seek care elsewhere. The avoidance of such challenging situations by continuing to prescribe ADT represents a barrier that might be, on the surface, addressed by providing discussion guides for providers. However, deeper psychological approaches addressing provider avoidance may also be considered.

In identifying barriers to and facilitators of de-implementation of low-value ADT, it should be noted that our provider sample engaged largely in facilitating behaviors despite our purposive sampling at the facility level raising challenges when pursuing residual low-value practices performed by few practitioners. The good news is that the barriers we identified are potentially modifiable. As a result, strategies can be designed to overcome these barriers based on the understanding of our study-derived conceptual model and the Behavior Change Wheel (e.g., targeting psychological capability for knowledge barriers, reflective motivation for beliefs about consequences barriers). Some approaches would likely need to be organizational, such as developing a menu of options for behavior change with respect to ADT prescribing (e.g., documenting informed consent), organizing collaborative and consultative bodies, providing guiding language, etc. With respect to patient perspectives on low-value ADT, our qualitative interviews raised themes consistent with prior studies [25, 26]. Mainly, a reliance on provider recommendations when it comes to prostate cancer treatment decision-making indicating provider-level strategies may have more potential in addressing low-value ADT in localized prostate cancer.

Our provider-level findings could underlie, at least in part, the differential de-implementation of four low-value breast cancer services selected by the Choosing Wisely campaign. For instance, significant de-implementation of axillary lymph node dissection and lumpectomy re-excision compared with stable to increasing rates of contralateral prophylactic mastectomy and sentinel lymph node dissection [30]. The former two also being at the tail end of de-implementation. In using the TDF to better understand barriers and facilitators across these low-value surgical cancer services, those already largely de-implemented, *knowledge* and *interpersonal skills* were similarly found as facilitators, as well as *social influence* and *group norms* akin to our findings of having multidisciplinary team input [5]. Taken together, further work using the TDF could facilitate a richer understanding across the spectrum of de-implementation.

Recognizing the provider distinctions proposed in our conceptual model is novel and important for several reasons. First, adoption is typically approached under the rubric of Rogers’ Diffusion of Innovations curve [31], whereas de-adoption is less well-characterized. The extent to which the diffusion categories (i.e., innovators, early adopters, early and late majority, laggards) and implications apply to de-adoption or de-implementation needs to be explored to advance science and facilitate de-implementation efforts. Second, recognizing whether the majority of providers are, or are not, participating in low-value care can appropriately direct inquiry efforts about the behavior of interest to be broad or quite focused. For instance, in our study, the majority of providers, despite our efforts to purposively sample high and low users, were not practicing low-value ADT, focusing our inquiry on perceptions of low-value castration and selected cases where it may be done, in addition to the discovery of the current state of guideline-based practices. This work sheds light upon the unique clinical indications beyond typical guideline considerations. Third, in seeking strategies to minimize low-value care, understanding if providers are “willing to prescribe under some circumstances” versus routinely find the practice “acceptable” will indicate the heavy lifting necessary to influence the latter group as the former group may need less justification for changing practice and already demonstrates decision-making concordant with patient values. Lastly, this model of provider characterization can add clarity to the specification of target audiences for behavior change strategies addressing low-value care [20, 32, 33]. Identifying distinct groups of providers with different attitudes to de-implementation is important in order to target or tailor complex health interventions [34] to address low-value care to those who would benefit from them without burdening those providers who do not need them (e.g., automated reminders, pre-authorization). Overall, we believe the respective proportions of providers across these broad categories are informative, alongside the extent of the practice gap, to guide de-implementation intervention selection and tailoring.

### Limitations

This study’s strengths rest upon the richness of data from providers across a national health system, and the contextualization of findings within existing theoretical models of behavior change, allowing for a contribution to the broader implementation science field. Several limitations should also be considered. First, despite an experienced qualitative team, there may be aspects not accounted for by our adherence to the concepts of the TDF and COM-B. On the other hand, using the concepts of the TDF and COM-B, we do provide a comprehensive look allowing us to operationalize concepts that can be considered in designing interventions aimed at behavior change. Second, studying the tail end of de-implementation is limited in that finding providers and sites practicing low-value care can be challenging as was found in this study. This may lead to limited inference with respect to barriers acting as behavior change targets and ultimately few providers needing to change practice to address remaining low-value care. Nonetheless, when low-value castration is initiated, often by other providers, we found providers tended to continue the practice. This opens up de-implementation opportunities even at this later phase given the long-term nature of the ongoing treatment. Moreover, better understanding late de-implementation may also have implications for the cost-effectiveness of interventions in this scenario, i.e., limiting it to care considered highly harmful. Third, while our experienced qualitative team did not identify any new TDF domains, we did find our consensus process helpful with respect to consistently coding data and aligning the findings according to a given TDF domain. We recognize there may be different interpretations of our findings in some cases (e.g., coding and implications of provider vs. patient emotion); however, the team has a deep knowledge of this area of care and has justified the interpretations throughout. Lastly, this study sample only included providers from a national health system removed from the fee-for-service pressures and financial incentives to deliver care. However, reimbursement for ADT decreased significantly in 2005 [14] minimizing financial incentives for low-value prescribing within and outside the system maintaining the broad relevance of our findings.

## Conclusions

Our use of behavioral theory-based methods to identify the most important barriers to and facilitators of decreasing remaining low-value ADT practices may inform theory-based de-implementation strategy development. Our conceptual model highlights relevant provider characteristics when it comes to considering the clinical and scientific impact of de-implementation strategies addressing low-value localized prostate cancer treatment with ADT. It may be relevant to address the tail end of low-value treatments for indolent cancer in general. At this point, ending the practice of low-value ADT for localized prostate cancer and other low-value indications will depend on both the development of strategies for further de-implementation and the prioritization of de-implementation by individual providers and institutions caring for patients with prostate cancer.

### Trial status

Provider recruitment for this work started in August 2018.

## Supplementary Information

**Additional file 1.**

## Data Availability

The data sets supporting the conclusions of this article will be shared upon request. Members of the scientific community who would like a copy of the final data sets (i.e., data sets underlying any publication) from this study can request a copy by e-mailing Dr. Ted Skolarus at Ted.Skolarus@va.gov. The investigator should state their reason for requesting the data and their plans for analyzing the data. De-identified data will be provided after requestors sign a Letter of Agreement detailing the mechanisms by which the data will be kept secure and access restricted to their study team. The agreements will also state the recipient will not attempt to identify any individual whose data are included and will not share the data with anyone outside of their research team. The data set will not include private or protected information, and all dates will be changed to integers to allow for the calculation of time periods. Final data sets will be copied onto a CD and limited data sets will be encrypted, and the password will be sent to the requestor via e-mail. The CD will be sent to the requestor via FedEx. Each data set will be accompanied by documentation that lists all variables described in the publication and links them with variable names in the data set.

## Abbreviations

ADT: Androgen deprivation therapy
AUA: American Urological Association
COM-B: Behavior Change Wheel’s Capability, Opportunity, Motivation Model
TDF: Theoretical Domains Framework
NCCN: National Comprehensive Cancer Network
MUSIC: Michigan Urological Surgery Improvement Collaborative
