# Correction: Learning from the “tail end” of de-implementation: the case of chemical castration for localized prostate cancer

**DOI:** 10.1186/s43058-023-00411-9

**Published:** 2023-03-20

**Authors:** Ted A. Skolarus, Jane Forman, Jordan B. Sparks, Tabitha Metreger, Sarah T. Hawley, Megan V. Caram, Lesly Dossett, Alan Paniagua-Cruz, Danil V. Makarov, John T. Leppert, Jeremy B. Shelton, Kristian D. Stensland, Brent K. Hollenbeck, Vahakn Shahinian, Anne E. Sales, Daniela A. Wittmann

**Affiliations:** 1 VA HSR&D Center for Clinical Management Research, VA Ann Arbor Healthcare System, Ann Arbor, MI USA; 2 Department of Urology, Dow Division of Health Services Research, University of Michigan Medical School, Ann Arbor, MI USA; 3 Department of Internal Medicine, University of Michigan Medical School, Ann Arbor, MI USA; 4 Rogel Cancer Center, Michigan Medicine, Ann Arbor, MI USA; 5 VA New York Harbor Healthcare System and NYU School of Medicine Departments of Urology and Population Health, New York, NY USA; 6 Surgical Service, VA Palo Alto Health Care System, Palo Alto, CA USA; 7 Department of Urology, Stanford University, Stanford, CA USA; 8 VA Greater Los Angeles Healthcare System, Los Angeles, CA USA; 9 Department of Learning Health Sciences, University of Michigan Medical School, Ann Arbor, MI USA

**Correction: Implement Sci Commun 2, 124 (2021)**

**https://doi.org/10.1186/s43058-021-00224-8**

Following the publication of the original article [1] the authors requested to update the “Competing interests” section as follows:

“The authors declare that Anne Sales is co-Editor-in-Chief of the journal."

## References

[1] Skolarus TA (2021). Learning from the “tail end” of de-implementation: the case of chemical castration for localized prostate cancer. Implement Sci Commun.

