# Supplementary material for: Learning from the “tail end” of de-implementation: the case of chemical castration for localized prostate cancer
Source: Implement Sci Commun. 2021 Oct 28;2:124. doi: 10.1186/s43058-021-00224-8 (PMC8555144; doi:10.1186/s43058-021-00224-8)
Supplement: Supplementary file 1 — Additional file 1. [file 43058_2021_224_MOESM1_ESM.docx]

# DeADT

# Interview Guide for Provider Semi-Structured Interview

# Providers who do NOT use low value ADT

**Introduction:**

Thank you for agreeing to participate in this interview. We are interested in learning how urologists practice with respect to androgen deprivation therapy (ADT). We know that physicians practice in different ways and we want to learn about that. During this interview, I will ask about your experience and opinions on using androgen deprivation therapy.

Your participation is purely voluntary and you may choose not to answer any questions. You will not be penalized in any way for deciding to stop participation.

The interview should take approximately 30-45 minutes and will be audio taped so that we don’t miss anything that you have to say. Any information you provide will be handled in a confidential manner. Your name will not appear on any of the transcripts. The interview transcript will be given a study ID number, and any personal references that would identify any individuals will be removed.

Do you have any questions? If you have questions later, you may contact a member of the study team at 1-800-753-3357 (option #2- for the *Prostate Cancer* study). Are you still interested in participating in this interview?

Great, I am going to turn on the audio recorder and will ask you to state your consent to be recorded, and then we will get started.

**Index patient:**

First, I’ll describe a hypothetical, typical patient (who might come to your practice) and ask you questions about how you would think of treating this patient.

Imagine a 72 y/o obese, white male with intermediate (for example, Gleason 7, and PSA 11) risk localized prostate cancer and no family history of prostate cancer. He has mild erectile dysfunction and minimal urinary symptoms. He does not want surgery or radiation therapy, and asks about hormone therapy to treat his cancer. Do you want me to repeat anything?

***Interviewer***: If participant asks, say that there are no scans in this scenario.

**Index Patient Questions**

1. Tell me your thoughts about how you would think about treating this patient.

***Interviewer****: Stay open; probe using participant’s language.*

***Listen for****: What are the most important factors that influence your decision-making? For example…*

- *Stage of cancer?*
- *Other health conditions?*
- *Severity of urinary symptoms?*
- *Cost of care?*
- *Patient satisfaction (where does it rank in consideration)?*
- *Peer behavior?*
- *Possible side effects?*
- *Guidelines?*
- *Past experience?*
- ***Would it matter at all if it was intermediate risk- but what if patient was high risk? Rapidly rising PSA or fast doubling time?*** *– ask later in interview if it doesn’t come up before.*
- *Anything else?*

**IF PROVIDER SAYS HE/SHE WOULD NOT USE ADT**

1. What are your own feelings about some providers using ADT with a patient like this?

***Probe****: Patients may think of it as a curative treatment, what do you think about that? How do you deal with that?*

***Interviewer:*** *Want to get at if OTHER providers think ADT will delay spread, lower PSA, etc.*

1. Can you lay out for me how you take a patient’s preference into account if the patient has one like in this example?
   - *What if patients were more insistent?*
   - *How do you approach that if they come to you and are already on ADT for localized prostate cancer?*

***Interviewer****: Feedback what we heard.*

**General Questions**

1. What kind of guidance do you use (for yourself) when making decisions about treating patients with ADT? In the scenario we gave you, and in other cases, too.

***Probe****: Requirements of your organization? By external organizations (e.g., Commission on Cancer; National Comprehensive Cancer Network, any guideline)?*

1. Instead of giving someone ADT for localized prostate cancer treatment, what could you offer them instead? e.g., surveillance/ watchful waiting, surgery, radiation?

***Probe***: *Specifically, what would you say to the patient to offer them a: PSA check every 6 months, diet or exercise program, intermittent ADT, etc.*

***Interviewer***: Want to know how they would get patients to do this.

1. In the Urology world, how is treating patients with primary ADT perceived? What about colleagues in your practice?

***Probe****: Do you think that you or providers who prescribe ADT are influenced by the way colleagues practice? Are there other influences that operate for providers who prescribe ADT? e.g., facility policy and culture, peers, patient preference?*

1. Is ADT something you talk about with your colleagues?
2. If you were to imagine yourself in the shoes of a patient with localized prostate cancer who was prescribed ADT, what comes to mind?

**Conclusion**: That’s all the questions I have for you today; is there anything else you would like to add that I have not asked your already?
